# P2RX1 Influences the Prognosis of Ph+/Ph-Like ALL through Energy and Calcium Metabolism

**DOI:** 10.32604/or.2025.068814

**Published:** 2025-12-30

**Authors:** Xiangmei Ye, Baoyi Yang, Xin Zhang, Luyuan Yang, Likun Zhang, Qin Ren, Xiaobing Li, Leiguang Feng, Lanlan Wei, Peng Song, Yuqing Ye, Xin Lian, Yujuan Gao, Haidi Tang, Zhiyu Liu

**Affiliations:** 1 Department of Laboratory Diagnostics, The First Affiliated Hospital of Harbin Medical University, Harbin, 150000, China; 2 Key Laboratory of Hepatosplenic Surgery, Ministry of Education, The First Affiliated Hospital of Harbin Medical University, Harbin, 150000, China; 3 Department of Pathogenic Microbiology, Harbin Medical University, Harbin, 150000, China; 4 Third Department of Surgery, The First Affiliated Hospital of Heilongjiang University of Traditional Chinese Medicine, Harbin, 150000, China; 5 Central Laboratory, The First Affiliated Hospital of Harbin Medical University, Harbin, 150000, China; 6 National Clinical Research Center for Infectious Diseases, The Third People’s Hospital of Shenzhen, Shenzhen, 518000, China; 7 The Second Hospital Affiliated to Southern University of Science and Technology, Shenzhen, 518000, China; 8 Guangzhou Xuekang Lu Daopei Biotechnology, Guangzhou, 510000, China; 9 Department of Hematology, The First Affiliated Hospital of Harbin Medical University, Harbin, 150000, China

**Keywords:** Philadelphia chromosome-positive acute lymphoblastic leukemia (Ph+ALL), Philadelphia chromosome-like B-cell acute lymphoblastic leukemia (Ph-like ALL), transcriptome sequencing, P2X purinoceptor 1

## Abstract

**Objectives:**

Philadelphia chromosome-positive B-cell acute lymphoblastic leukemia and Philadelphia-like B-cell acute lymphoblastic leukemia (Ph+/Ph-like ALL) constitute the majority of relapsed/refractory B-ALL (R/R B-ALL) cases, highlighting an urgent need to discover new therapeutic targets. This study aims to elucidate the mechanisms underlying poor prognosis in Ph+/Ph-like ALL through transcriptome sequencing and functional cytological assays, with the goal of informing new clinical treatment strategies.

**Results:**

Transcriptomic analysis of Ph+/Ph-like ALL patients revealed that low expression of P2X Purinoceptor 1 (P2RX1) was associated with unfavorable outcomes. Specifically, patients with poor prognosis and low P2RX1 expression exhibited downregulation of genes involved in energy and calcium metabolism pathways, along with upregulation of genes governing key cellular processes such as cell proliferation (e.g., MYC), cell cycle progression (e.g., CCND2), and apoptosis inhibition (e.g., DASP6). Cellular experiments demonstrated that SUP-B15 cells overexpressing P2RX1 displayed elevated intracellular levels of ATP, calcium, and glucose, together with enhanced glycolytic capacity, compared to empty vector controls. Treatment of SUP-B15 cells with dexamethasone (Dex), Imatinib, or their combination significantly suppressed proliferation and promoted apoptosis, which was accompanied by increases in intracellular ATP, calcium, and glucose. Moreover, exogenous ATP administration (a P2RX1 agonist) enhanced apoptosis and inhibited proliferation in control cells. Conversely, treatment with NF449 (a P2RX1 inhibitor) increased proliferation in both P2RX1-overexpressing and control SUP-B15 cells.

**Conclusion:**

Our findings indicate that P2RX1 may exert this function through modulating energy metabolism and calcium homeostasis, resulting in elevated intracellular calcium levels. Sustained elevation of calcium promotes apoptosis, whereas exogenous ATP activates P2RX1, enhances calcium influx, and attenuates the suppression of apoptosis associated with P2RX1 underexpression, ultimately correlating with improved treatment response.

## 1 Introduction

B-cell acute lymphoblastic leukemia (B-ALL) is a hematological malignancy originating from B-lineage progenitor cells. It is characterized by a differentiation arrest and maturation blockage at an early developmental stage, typically the precursor phase, leading to the clonal expansion of malignant lymphoid precursors in the bone marrow. The disease is driven by diverse genetic alterations, including gene mutations and chromosomal abnormalities, which directly dictate its biological behavior, risk stratification, therapeutic strategies, and prognosis [1]. B-ALL is not a single disease but a group of diseases with high molecular genetic heterogeneity, including over twenty subtypes [2]. The prognosis of B-ALL subtypes varies considerably and is dictated by a combination of clinical factors (e.g., age, white blood cell count) and biological features (e.g., cytogenetic abnormalities, fusion genes, and specific gene mutations) [3]. In accordance with the 2025 National Comprehensive Cancer Network (NCCN) Guidelines, B-ALL is classified into the following risk categories: Standard Risk: including such as high hyperdiploidy and ETV6::RUNX1 subtypes; High Risk: TCF3::PBX1 and BCR::ABL1 (Ph+) subtypes; Very High Risk: BCR::ABL1-like (Ph-like) and KMT2A-rearranged subtypes [4].

Recent years have witnessed remarkable progress in both therapeutic modalities and diagnostic technologies, transforming outcomes for BCP-ALL subgroups. The 5-year overall survival rate has now improved dramatically from under 50% to 75% [5]. Despite these advances, relapse and refractoriness remain the primary obstacles to achieving favorable long-term prognosis in B-ALL.

Philadelphia chromosome-positive B-ALL (Ph+ALL) is defined by the t(9;22)(q34;q11) translocation, this genetic alteration gives rise to the BCR-ABL1 oncogene, whose protein product is a dysregulated tyrosine kinase that sustains the activation and proliferation of leukemic cells [6]. The incidence of Ph+ALL exhibits a significant age-dependent increase. While it accounts for only about 5% of childhood/adolescent ALL cases, its prevalence rises to approximately 25% in adults and exceeds 50% in ALL patients over 50 years of age [7]. Nevertheless, Ph+ALL is classified under the poor prognosis group according to NCCN 2021 guidelines [8]. Using Tyrosine Kinase Inhibitors (TKIs), many patients can achieve remission status on the first treatment; however, a significant number of patients still experience relapse and drug resistance [9].

The Philadelphia chromosome-like (Ph-like) ALL subtype is characterized as a distinct high-risk subgroup. It exhibits a gene expression signature analogous to BCR-ABL1-positive disease but in the absence of the underlying translocation [10,11]. Ph+ALL and Ph-like ALL (Ph+/Ph-like ALL) share a profile of high-risk clinical features, such as elevated white blood cell (WBC) counts, a higher rate of failure to achieve remission with standard induction therapy, higher levels of measurable residual disease, and lower survival rates, which establishes them as the primary subtypes of refractory B-ALL [12].

Several pioneering studies have described the potential biological pathways of selective pressure and supportive therapy failure by analyzing paired B-ALL diagnosis/relapse samples. These studies have identified not only the genetic and epigenetic alterations in leukemia associated with prognosis, but also clonal evolution models based on tumor heterogeneity [13–15]. However, the mechanism of the recurrence and refractoriness of Ph+/Ph-like ALL is not quite clearly understood. Therefore, we utilized transcriptome sequencing coupled with cytology experiments and bioinformatics methods to analyze the molecular biological features that are closely associated with the onset and progression of recurrence and refractoriness of Ph+/Ph-like ALL, and to screen for molecular markers of recurrence and refractoriness of Ph+/Ph-like ALL, which would provide a molecular biological basis for the prognostic risk stratification and for the individualization of treatment. Future studies must aim to further identify and screen more Ph+/Ph-like ALL patients, who are potential candidates for targeted molecular therapies, which is essential for developing targeted therapies and promoting precision medicine.

## 2 Patients and Methods

### 2.1 Patients

A total of 18 patients with B-cell acute lymphoblastic leukemia (B-ALL), initially diagnosed and treated in the Department of Hematology at the First Affiliated Hospital of Harbin Medical University from January 2019 through December 2023, were included in this study. The cohort included 3 cases of poor prognosis Not-Ph+/Ph-like and 15 cases of Ph+/Ph-like ALL. There were 10 male patients with a mean age of 31.8 ± 3.89 years and 8 female patients with a mean age of 39.62 ± 3.04 years. All cases were diagnosed according to the MICM criteria (Morphology, Immunology, Cytogenetics, and Molecular biology). Bone marrow samples were collected from three paired primary/relapsed Not-Ph+/Ph-like B-ALL and three paired primary/relapsed cases of Ph+/Ph-like B-ALL patients, all of which exhibited poor prognosis. For the remaining cases, separate initial bone marrow samples were collected.

The criteria for patient recurrence/relapse include: I. Relapsed ALL: Defined as the recurrence of the disease after achieving complete remission (CR), regardless of the time of occurrence. Specifically, it includes: (1) Bone marrow recurrence: Presence of >5% of primitive lymphocytes in the bone marrow (and no other cause can explain it), or the emergence of new extramedullary diseases. (2) Extramedullary recurrence: Occurring in the central nervous system (CNS), testicles, eyes, or other parts. II. Refractory ALL: Mainly refers to the failure to achieve remission at the end of induction chemotherapy. After the completion of standard induction chemotherapy (approximately 4 weeks), if no complete remission (CR) is achieved, that is, the primitive lymphocytes in the bone marrow are still ≥5%, or extramedullary diseases persist.

This research was approved by the Ethics Committee of the First Affiliated Hospital of Harbin Medical University (Approval No.: IRB-AF/SC-04/02.0) and was conducted in accordance with the principles of voluntary and informed consent. All waste blood samples and clinical data collected met the required ethical standards.

### 2.2 RNA Sequencing (RNA-Seq) Assay

Total mRNA was isolated from thawed human bone marrow mononuclear cells (AllPrep DNA/RNA/Protein Mini Kit (80004), QIAGEN, Shanghai, China), cells were lysed in Buffer RLT, and the lysate was processed through an AllPrep DNA spin column. The flow-through was ethanol-adjusted and applied to an RNeasy spin column for RNA purification, which involved a series of washes (Buffers RW1 and RPE) followed by elution in RNase-free water. RNA concentration and purity (A260/A280) were assessed on a NanoDrop 1000 spectrophotometer. Subsequently, 1 μg of total RNA was reverse-transcribed into cDNA (Reverse Transcription System(A3500), Promega, Madison, Wisconsin, USA). Finally, sequencing libraries were constructed and analyzed on an Illumina HiSeq 2000 platform (TruSeq SBS chemistry (FC-401-1001), Illumina, San Diego, CA, USA).

### 2.3 Stable Transfer Cell Line Establishment and Drug Treatment

Stable P2RX1-overexpressing and empty vector control SUP-B15 cell lines were established using lentiviral transduction (lentiviral; GeneChem Co, Ltd., Shanghai, China). The SUP-B15 parental cell line was kindly provided by Professor Ce Shi (Central Laboratory, The First Affiliated Hospital of Harbin Medical University, Harbin, China). Cells were cultured in Iscove’s Modified Dulbecco’s Medium (IMDM; Gibco, Life Technologies, Carlsbad, CA, USA) supplemented with 20% fetal bovine serum (FBS; ExCell Bio, Suzhou, China) and 1% penicillin-streptomycin (Beyotime Biotechnology, Shanghai, China) at 37°C in a 5% CO_2_ atmosphere [16].

Treatment group setup and drug addition: blank wells, 50 μmol/L ATP group (ATP; Beyotime Biotechnology, Shanghai, China), (0, 0.5, 5, 500, 5000, 25,000) nmol/L NF449 (a highly efficient and selective competitive antagonist of the P2RX1 receptor) group (MedChemExpress, Shanghai, China), 0.25 μmol/L Dexamethasone group (Dex; Pujin Linzhou pharmacy Co., Shanghai, China), 30 μmol/L Imatinib group (Imatinib; MedChemExpress, Shanghai, China), and the combined treatment group of 0.25 μmol/L Dex+ 30 μmol/L imatinib.

### 2.4 CCK8 for Cell Proliferation Assays

We plated cells in 96-well plates (5 × 10^4^ cells/well) and treated them with various compounds for 24, 48, and 72 h to evaluate their effects. Cell proliferation inhibition was evaluated using a Cell Counting Kit-8 (CCK-8; C0038, Beyotime Biotechnology, Shanghai, China). The absorbance was measured using a SpectraMax iD5 multi-mode microplate reader (Molecular Devices, San Jose, CA, USA).

### 2.5 Apoptosis Detection by Flow Cytometry

After 24 h of drug treatment, 5 × 10^5^ cells were collected. Following the manufacturer’s instructions, 2.5 μL of both PI-and APC-conjugated antibodies were added. Cell apoptosis was assessed by Lyric flow cytometry (BD Biosciences, San Jose, CA, USA) according to the manufacturer’s instructions of the apoptosis assay kit (Elabscience, E-CK-A211, Wuhan, China).

### 2.6 Reverse Transcription qPCR (qRT-PCR) Method for Gene Expression Detection

P2RX1 and MCM5 expression was assessed by qRT-PCR (GAPDH reference) using the 2^−ΔΔCT^ method. The 20 μL reaction mix included TB Green Premix, primers (Table A1) (Thermo Fisher Scientific, Waltham, MA, USA), and cDNA. Cycling conditions comprised initial denaturation (95°C, 30 s), 40 cycles (95°C, 5 s; 56°C, 30 s), and melting curve analysis (TB Green^®^ Premix Ex Taq™ II; Takara Bio Inc., Kyoto, Japan).

### 2.7 Dynamic Fluorescence Detection of Oxygen Consumption Rate (OCR) and the Extracellular Acidification Rate (ECAR)

At a density of 1.5 × 10^6^ cells per well, cells were seeded into 6-well plates. After 24 h of treatment with various drugs, the cells were subjected to metabolic stress for 6 h with the following compounds: 10 μM FCCP (MedChemExpress, Shanghai, China), 100 μM antimycin A (AA), 10 mM high glucose (Glu), or 5 mM 2-deoxy-D-glucose (2-DG) (AA, Glu, and 2-DG, Sigma-Aldrich, St. Louis, MO, USA). We then plated cells for real-time metabolic analysis in 96-well plates at 5 × 10^5^ cells per well. Cellular metabolic function was evaluated by measuring the OCR and ECAR via a multi-functional microplate reader (SpectraMax iD5, Molecular Devices, San Jose, CA, USA). The instrument settings were as follows: The detection mode was set to Time-Resolved Fluorescence, with excitation and emission wavelengths of 380 and 650 nm, respectively. A kinetic read was performed over a total duration of 120 min, with measurements taken at 3-min intervals. Orbital shaking was enabled prior to each reading to ensure sample homogeneity. The temperature was maintained at 37°C, and the plate was pre-incubated within the instrument for 15 min before initiating the assay to achieve thermal equilibrium across all wells. Following the run, the fluorescence intensity-time curve for each well was generated, and the slope of this curve was calculated to represent the relative OCR or ECAR (OCR: BB-48211 and ECAR: BB-48311, BestBio, Shanghai, China).

### 2.8 Detection of ATP, Ca^2+^, and Glu Concentrations by Chemiluminescent Techniques

At 24 and 48 h post-drug treatment, cells were harvested. Subsequently, cell lysis was performed separately on ice with 200 μL of ATP lysis buffer and Ca^2+^ lysis buffer, respectively. Intracellular concentrations of ATP, Ca^2+^, and glucose (Glu) were measured according to the manufacturer’s protocols. The concentrations were quantified using standard curves derived from the following kits: ATP Assay Kit (Beyotime Biotechnology, S0026, Shanghai, China); Calcium Ion (Solarbio, S1063, Beijing, China) and Glucose Assay Kits (Nanjing Jiancheng, A154-2-1, Nanjing, China).

### 2.9 Bioinformatic Analysis

The number of mRNA reads obtained was normalized by transcripts per million (TPM). Prior to RNA-Seq analysis, lowly expressed genes (e.g., those with TPM < 1) were filtered out to reduce the burden of multiple testing. We utilized the HG19 build as the reference genome. Processing of the raw data was conducted in the R environment (R 4.3.3), and identified differentially expressed genes using the ‘LIMMA’ package (3.58.x); *p* < 0.05 and |log2FC| ≥ 1 were set to be statistically significant. A volcano plot was constructed to summarize the output, highlighting genes that were both statistically significant and exhibited large-magnitude changes. We annotated and enriched the gene biological functions and underlying pathways using Gene Ontology (GO) and Kyoto Encyclopedia of Genes and Genomes (KEGG). Illumina DRAGEN software (DRAGEN v3.10.x) was utilized for mutation calling and analysis. Visualize the overlap of differentially mutated gene sets via a Venn diagram. Protein-protein interaction (PPI) networks for the candidate genes were developed and validated using GeneMANIA (http://www.genemania.org). The clustering outcome was visualized in a two-dimensional space using principal component analysis (PCA).

### 2.10 Statistical Analysis

We performed statistical analysis and data graphing using SPSS (version 25.0.0.2; IBM, Armonk, NY, USA) and GraphPad Prism (version 8.0.2(263); GraphPad, San Diego, CA, USA), respectively, and using chi-square for counts and mean± standard deviation (SD) for measures. Group differences were analyzed with one-way ANOVA, and Kaplan-Meier analysis was used to evaluate survival. *p*-values were derived from a one-sided unstratified log-rank test, considering a *p*-value < 0.05 as statistically significant (n = 3 independent experiments per group).

## 3 Results

### 3.1 General Clinical Characteristics of Patients

Fifteen Ph+/Ph-like ALL patients were divided into a good (n = 5) and a poor (n = 10) group according to the therapeutic effect. To investigate the heterogeneity of clinical characteristics in both groups, we conducted comparative analyses of White Blood Cell (WBC), Patelet (PLT), Haemoglobin (Hb), bone marrow blast, Minimal Residual Disease at Day 15 (MRD15), Minimal Residual Disease at Day 33 (MRD33), C-reactive protein (CRP), and calcitonin (CT) levels. The statistical comparison demonstrated no significant differences between the two groups (*p* > 0.05). Only the Overall Survival (OS) was different (*p* = 0.03) (Table 1).

**Table 1:** Statistical analysis of clinical characteristics of Ph+/Ph-like ALL patients

Variable	Ph+/Ph-like	Ph+/Ph-like	χ^2^/F	*p*
	(Good N = 5)	(Poor N = 10)		
**Sex (%)**			−1.73	0.13
F	40.00 (2/5)	40.00 (4/10)		
M	60.00 (3/5)	60.00 (6/10)		
**Age (year)**	17.41 ± 26.14	40.12 ± 20.18	3.65	0.61
**WBC (10** ^**9**^ **/L)**	111.88 ± 173.56	55.29 ± 69.17	0.70	0.51
**Hb (g/L)**	70.61 ± 33.96	94.14 ± 29.18	0.07	0.79
**PLT (10** ^**9**^ **/L)**	54.12 ± 81.10	39.31 ± 29.66	0.39	0.71
**BM blasts (%)**	83.29 ± 4.50	73.46 ± 24.79	1.44	0.18
**MRD% (15 day)**	1.62 ± 0.24	3.18 ± 0.31	−2.11	0.10
**MRD% (33 day)**	0.04 ± 0.05	1.52 ± 0.30	−1.00	0.39
**OS**	22.10 ± 4.57	7.00 ± 8.27	2.89	0.03*
**CRP (mg/L)**	65.12 ± 46.03	64.68 ± 56.72	0.01	0.99
**CT (mg/L)**	0.24 ± 0.04	0.75 ± 1.02	−1.39	0.21

Note: Data are presented as median (interquartile range) for continuous variables and n (%) for categorical variables; Ph+/Ph-like, Ph+ALL and Ph-like ALL; WBC, white blood cell count; Hb, hemoglobin; PLT, platelet; BM, bone marrow; MRD, minimal residual disease; OS, overall survival; CRP, C-reactive protein; CT, chemotherapy, *p < 0.05.

### 3.2 Ph+/Ph-Like ALL Gene Mutation Analysis

**Figure 1:**
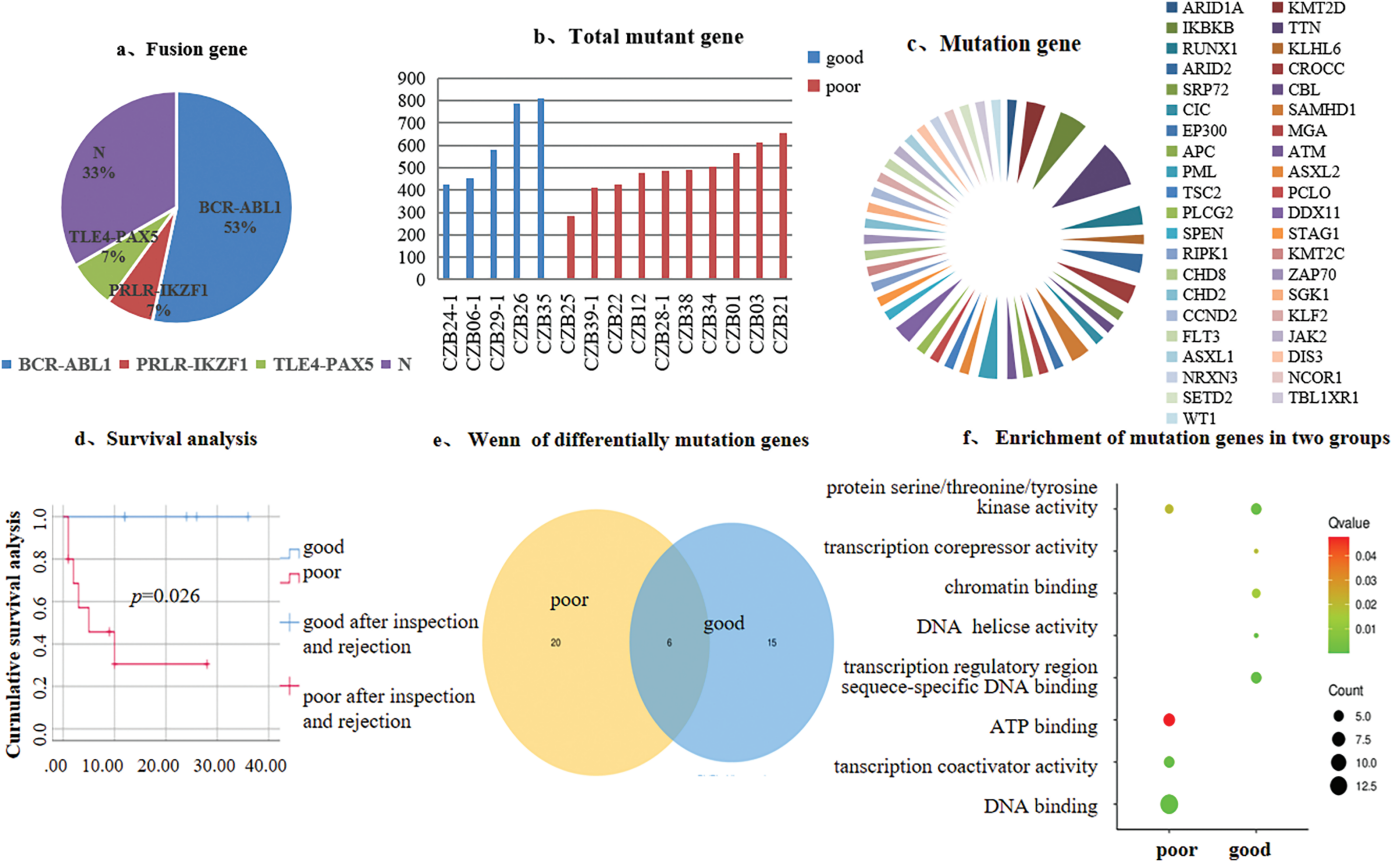
Genetic background of 15 Ph+/Ph-like ALL patients and mutation analysis. (**a**). Distribution of fusion genes; (**b**). Total mutant gene; (**c**). Disease-associated gene mutations; (**d**). Survival analysis; (**e**). Venn diagram of differentially mutated genes; (**f**). Mutation genes enrichment analysis

We analyzed transcriptome sequencing data from 15 Ph+/Ph-like ALL, which revealed diverse genetic alterations. Three types of fusion genes were detected: BCR-ABL1, TLE4-PAX5, and PRLR-IKZF1 (53%, 7%, and 7%) (Fig. 1a). Profile of the total mutated genes and disease-associated gene mutations in patients stratified by good vs. poor prognosis (Fig. 1b,c). Survival analysis was performed for both groups (*p* < 0.05) (Fig. 1d). There were a total of 41 disease-related mutant genes, among which six genes are shared (Fig. 1e). In the good prognosis group, enrichment analysis for disease-specific mutations identified significant association with biological processes including transcriptional co-repressor activity and DNA de-helicalization. In contrast, the poor prognosis group was in the pathways of ATP-binding and transcriptional co-activator activity (Fig. 1f). Although significant differences in survival outcomes were observed between the prognosis groups, but no statistically significant differences were found in genetic background characteristics (e.g., the number of SNP genes/loci, disease-related mutations/loci, and total mutant genes/loci; *p* > 0.05) (Table 2).

**Table 2:** Comparative analysis of genomic alterations between good and poor-prognosis Ph+/Ph-like patients

Variable	Ph+/Ph-like	Ph+/Ph-like	χ^2^/F	*p*
	(Good N = 5)	(Poor N = 10)		
**SNP gene**	50.20 ± 3.34	45.88 ± 5.44	1.83	0.09
**SNP locus**	73.81 ± 4.71	84.66 ± 16.93	−1.80	0.10
**Disease-related gene mutations**	4.82 ± 1.32	3.94 ± 2.02	1.04	0.32
**Disease-related gene mutations locus**	5.21 ± 1.30	4.40 ± 1.69	0.94	0.37
**Total mutant gene**	526.40 ± 191.06	405.56 ± 92.17	1.33	0.24
**Total mutant gene locus**	611.41 ± 181.28	500.33 ± 108.14	1.25	0.26

Note: Ph+/Ph-like, Ph+ ALL and Ph-like ALL; SNP, Single Nucleotide Polymorphism.

### 3.3 Ph+/Ph-Like ALL Gene Expression Analysis

**Figure 2:**
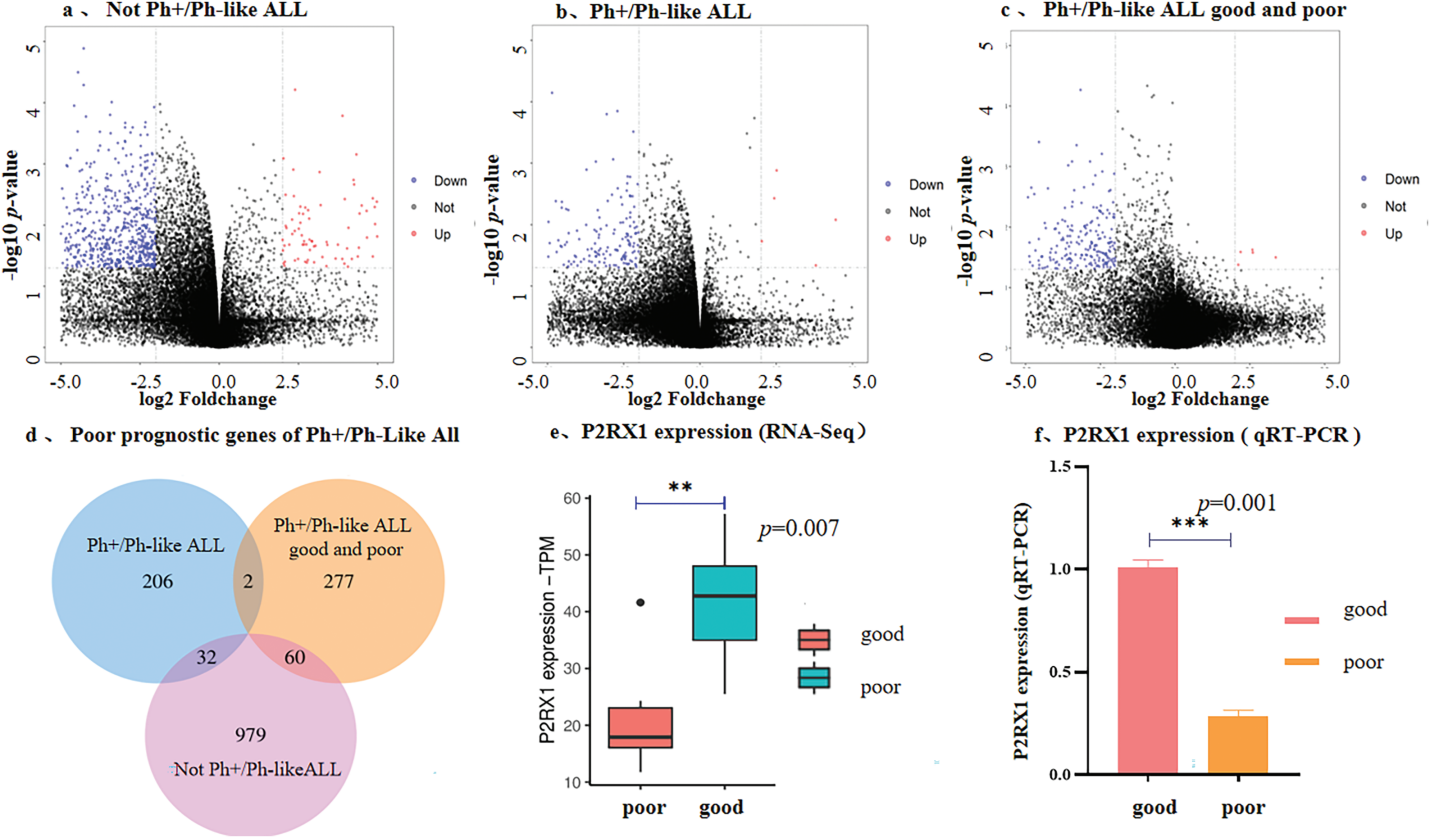
Correlation between low expression of P2RX1 and poor prognosis of Ph+/Ph-like ALL. (**a**). Volcano maps of DGEs from paired samples in Not-Ph+/Ph-like ALL; (**b**). Volcano maps of DGEs from paired samples in Ph+/Ph-like ALL; (**c**). Volcano maps of DGEs from newly diagnosed samples in Ph+/Ph-like B-ALL with good and poor prognosis (red dots indicate up-regulatory genes, blue dots indicate down-regulatory genes); (**d**). Venn diagram of DEGs in three groups; (**e**). P2RX1 expression in Ph+/Ph-like ALL (RNA-Seq); (**f**). P2RX1 expression in Ph+/Ph-like ALL (qRT-PCR). **p < 0.01, ***p < 0.001

To investigate the impact of differentially expressed genes (DEGs) on the prognosis of Ph+/Ph-like ALL, we analyzed the expression profiling data on paired primary/relapse bone marrow samples. The results showed that in the Not-Ph+/Ph-like ALL cohort (n = 3), 1071 DEGs were identified (140 upregulated and 931 downregulated) (Fig. 2a). In the Ph+/Ph-like ALL cohort (n = 3), 240 DEGs were identified (16 upregulated and 224 downregulated) (Fig. 2b). Notably, the proportion of downregulated genes was significantly higher in the Ph+/Ph-like ALL than in the Not-Ph+/Ph-like ALL (93.33% vs. 86.92%). Additionally, we analyzed RNA-Seq data from prognostically stratified Ph+/Ph-like ALL samples (Good Prognosis vs. Poor Prognosis) and identified 339 DEGs. Among these, only 9 genes were upregulated in the poor prognosis group, while 330 genes (97.35%) were downregulated (Fig. 2c).

To identify the genes associated with the poor prognosis of Ph+/Ph-like ALL, we performed a cross-cohort analysis using Venn diagrams. Two genes (P2RX1 and MCM5) were independently associated with a poor prognosis of Ph+/Ph-like ALL (Fig. 2d). RNA-Seq and qRT-PCR confirmed significantly lower P2RX1 mRNA expression in the poor prognosis group (Fig. 2e,f), but MCM5 showed no difference (*p* > 0.05).

The P2RX1 gene is an ATP-activated calcium channel that plays a significant role in maintaining intracellular calcium homeostasis, and calcium ions are involved in several biological processes, including cell proliferation, and immunity, and apoptosis, as a second messenger. An integrated analysis of mutant gene enrichment in ATP-binding pathways and differential expression profiling, identifying P2RX1 downregulation in the poor prognosis cohort, suggested that P2RX1 dysfunction may critically contribute to leukemogenesis and adverse clinical outcomes in Ph+/Ph-like ALL.

**Figure 3:**
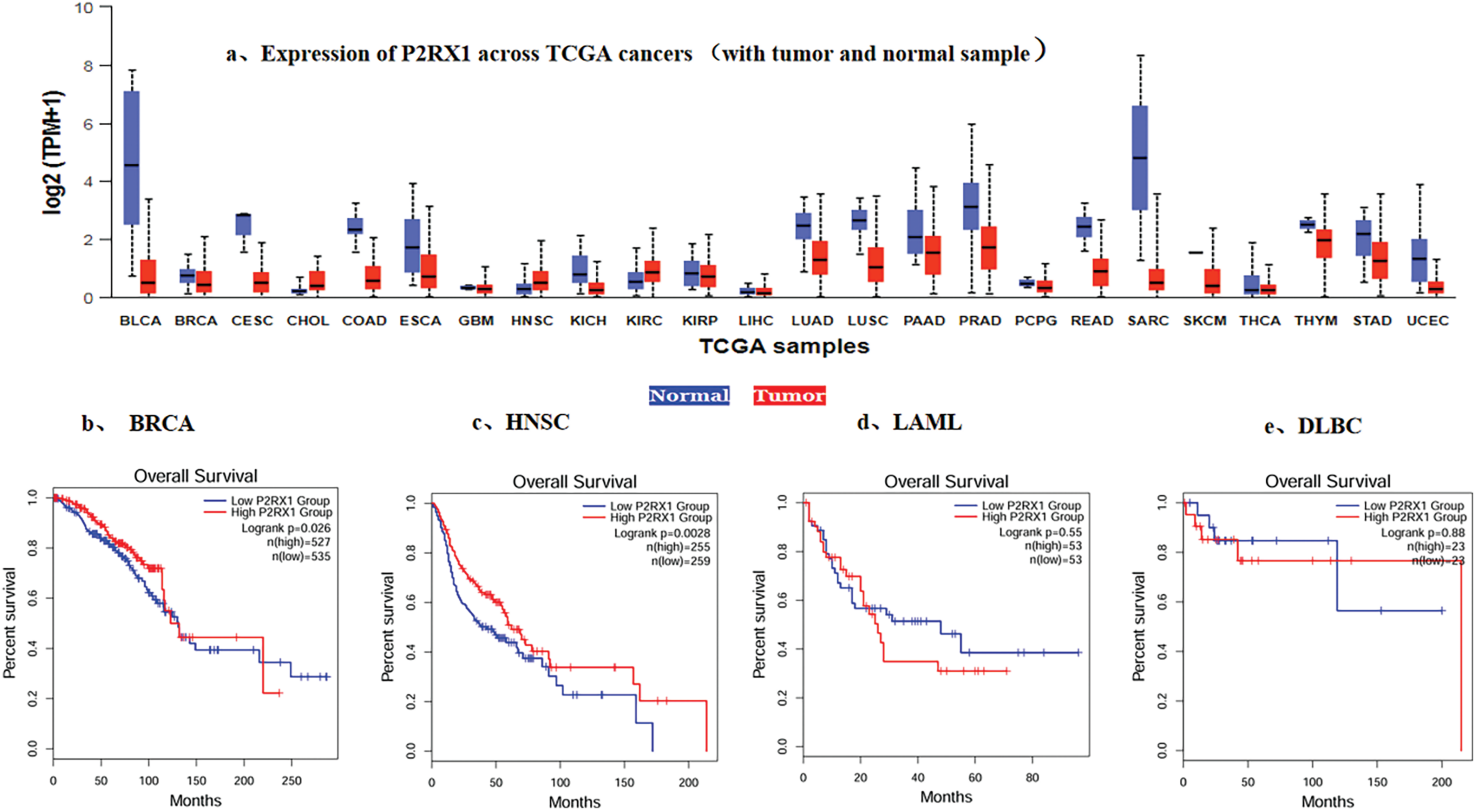
Expression of P2RX1 and effect of expression level on survival in Various types of Cancer. (**a**). Expression of P2RX1 across TCGA cancers (with tumor red and normal samples blue); (**b**). Effect of P2RX1 expression level on BRCA patient survival; (**c**). Effect of P2RX1 expression level on HNSC patient survival; (**d**). Effect of P2RX1 expression level on LAML patient survival; (**e**). Effect of P2RX1 expression level on DLBC patient survival

To verify the association between Overexpression of P2RX1 and good prognosis of tumors, we conducted a pan-cancer analysis using the Cancer Genome Atlas (TCGA) database, and the analysis of RNA-Seq data from 24 types of tumor tissues and their normal tissue samples showed that except for CHOL and HNSC, the P2RX1 expression in various tumors was lower than that of the corresponding normal tissues (Fig. 3a), and Overexpression was associated with a favorable clinical outcome in some tumors, such as BRCA and HNSC (Fig. 3b,c). However, the P2RX1 gene showed no association with AML and DLBC prognoses (Fig. 3d,e).

**Figure 4:**
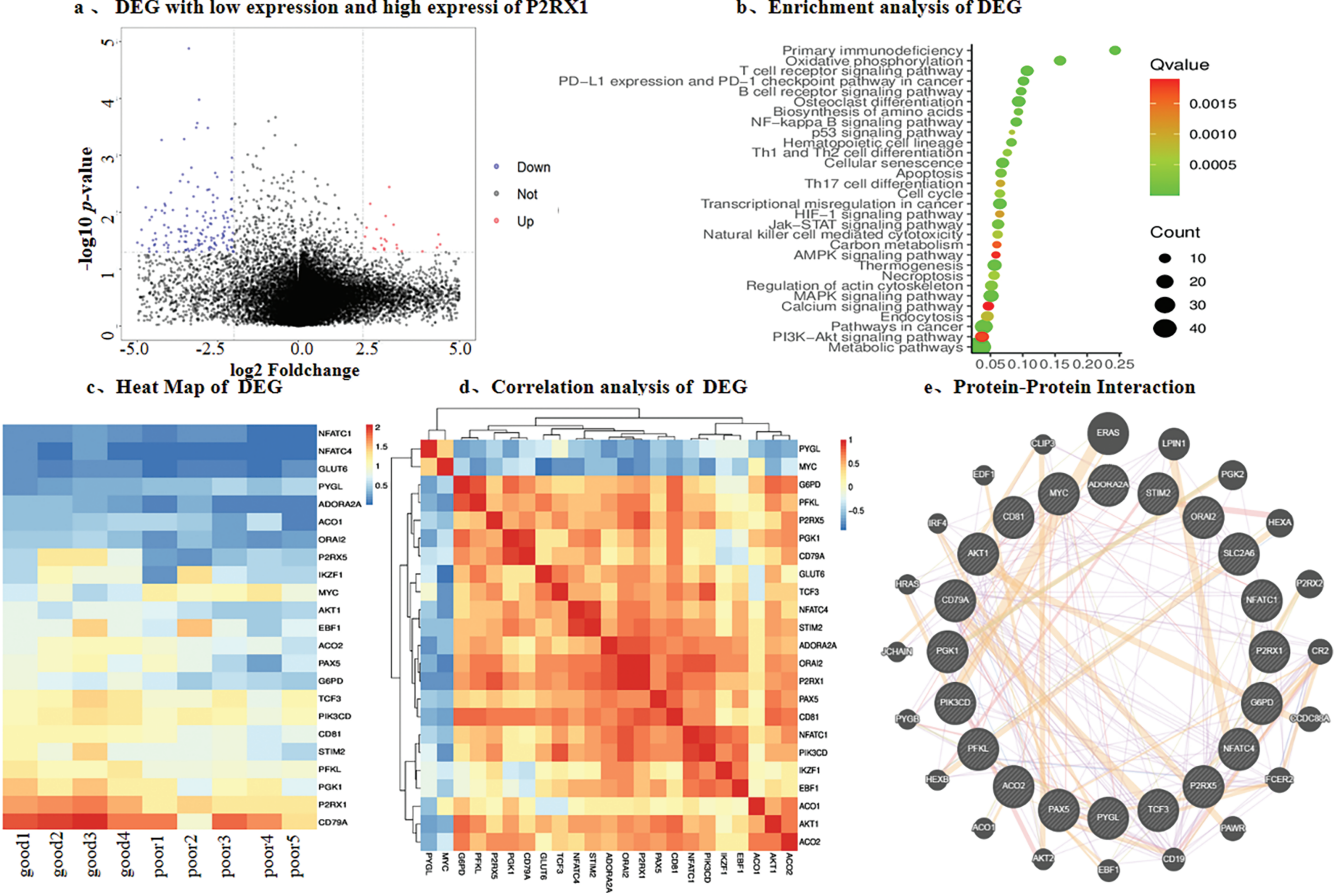
Differential gene analysis was performed according to the expression of P2RX1. (**a**). Differential gene volcano map of tumor cells above 80% and P2RX1 high expression with good prognosis group and P2RX1 low expression with the poor prognosis group; (**b**). Differential gene enrichment pathway analysis; (**c**). heat map of calcium metabolism differential, energy metabolism gene and B cell differentiation gene; (**d**). differential gene correlation analysis; (**e**). protein interaction analysis of differential gene

Therefore, we chose P2RX1 as an entry point for the poor prognostic mechanism of Ph+/Ph-like ALL. In order to prevent the possible influence of the tissue-of P2RX1 and reanalyzed 9 patients with tumor cell load higher than 80% at the time of initial diagnosis, among 4 patients with good prognosis and P2RX1 expression higher than the mean, 5 patients with poor prognosis and P2RX1 expression lower than the mean. A total of 592 DEGs were found, among which 93.92% were downregulated and only 6.08% of the genes were upregulated in the poor prognosis group (Fig. 4a). The enrichment analysis of the downregulated genes showed that many pathways related to tumorigenesis and development, such as immune pathways, have T-cell and B-cell receptor signaling pathways. In addition, metabolism-related pathways were enriched in the carbon metabolism, calcium metabolism pathway, thermogenesis, and AMPK signaling pathway. Pathways related to cell death were enriched in the apoptosis pathway (Fig. 4b). Despite enrichment analysis of upregulated genes, no pathway was enriched. However, MYC and CCND2 were involved in cell cycle blockade, negative regulation of apoptosis, positive regulation of proliferation, and cellular response to hypoxia, while SLC39A10 was linked to positive regulation of B-cell proliferation.

The P2RX1 gene connects energy metabolism and immunity, so we investigated the energy metabolism genes and found that the genes related to glucose transport, the tricarboxylic acid cycle, and the glycolysis pathway, such as GLUT6, ADORA2A, ACO1, G6PD, PGK1, and PFKL, had different degrees of low expression. In contrast, the PYGL gene related to glycogenolysis was highly expressed. The functional pathways of P2RX1, such as the calcium metabolism genes ORAI2, STIM2, and P2RX1, affected B cell differentiation genes IKZF1, EBF1, PAX5, TCF3, CD79A, CD81, and apoptosis-related genes, all of which showed low expression in the poor prognosis group (Fig. 4c). Moreover, protein-protein interaction network analysis was used to find out that P2RX1 genes were interrelated with these genes and the MYC oncogene (Fig. 4d,e).

### 3.4 P2RX1 Gene Overexpression Affects Energy Metabolism and Ca^2+^ Metabolism in Ph+ALL Cell Lines

**Figure 5:**
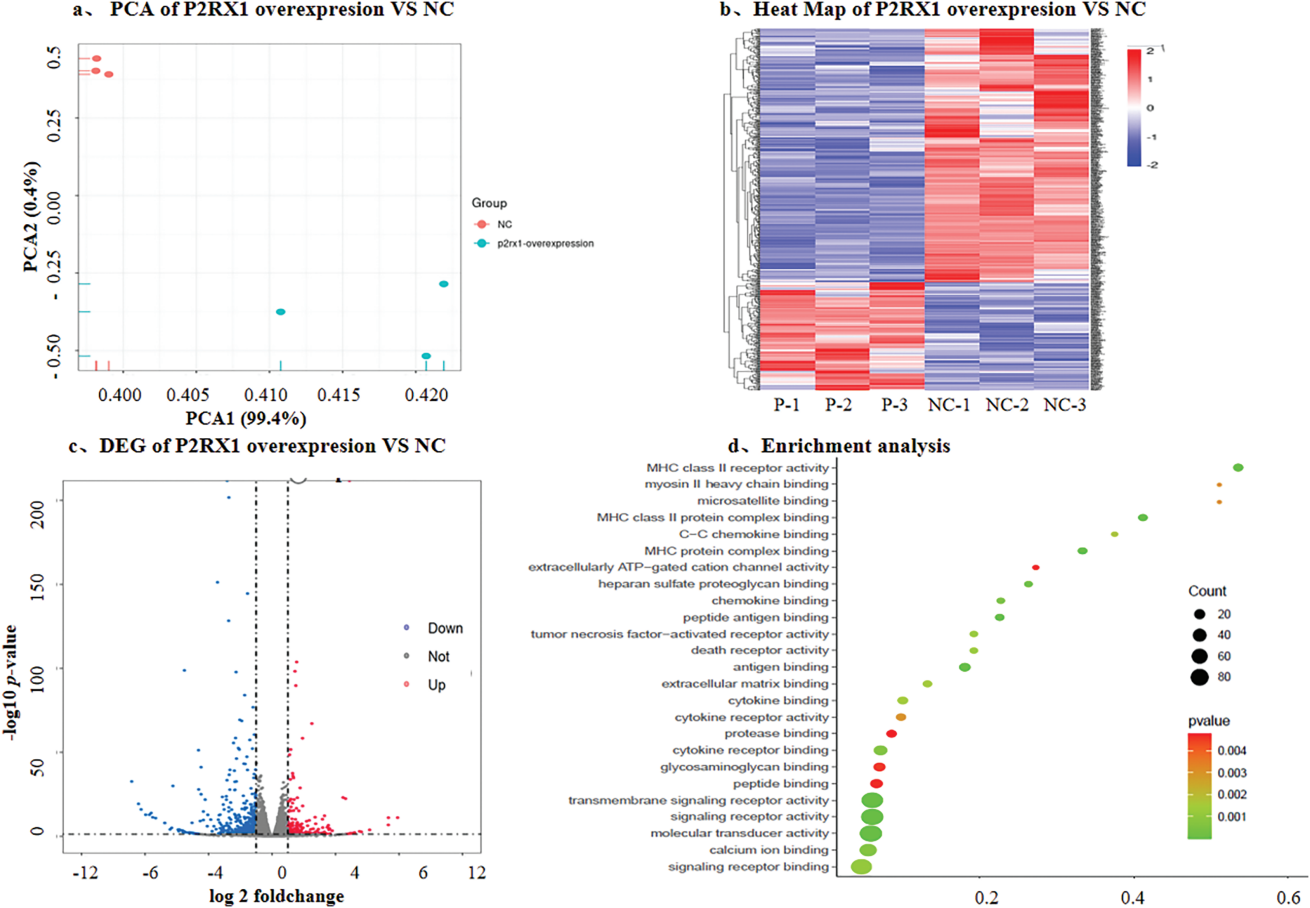
DEG with overexpresson P2RX1 and NC in SUP-B15 cell. (**a**). PCA of P2RX1 overexpresson and NC; (**b**). Heat map of P2RX1 overxpresion vs. NC; (**c**). Volcano maps of differentially expressed genes; (**d**). Enrichment analysis

In order to further investigate the possible mechanism of P2RX1 gene overexpression and Ph+/Ph-like ALL prognosis, we constructed the P2RX1 plasmid, utilized lentiviral infection to establish the P2RX1 gene overexpression and null viral vector control group (NC) stably transfected with the SUP-B15 cell, and conducted transcriptome sequencing. The results of the differential gene screening and pathway enrichment showed that there were 627 DEGs, of which 189 genes were downregulated and 438 genes were upregulated (Fig. 5a–c), and the proportion of downregulated genes was significantly reduced compared with that of DEGs in the patients with good and poor prognosis groups (30.14% vs. 97.35%). The differential gene enrichment analysis revealed that pathways such as ATP-dependent ion channels, calcium binding, death receptor, and tumor necrosis factor-activated receptor pathways were activated in P2RX1 high SUP B15, which were correlated with good tumor prognosis (Fig. 5d). This finding provides a mechanistic link to the favorable prognosis observed in patients with high P2RX1expression.

The P2RX1 receptor is an ATP-gated cation channel, and a high expression of P2RX1 promotes the inward flow of extracellular Ca^2+^, resulting in increased cytosolic calcium concentrations. We hypothesized that the P2RX1 gene may affect the prognosis of Ph+/Ph-like ALL through calcium and energy metabolism.

**Figure 6:**
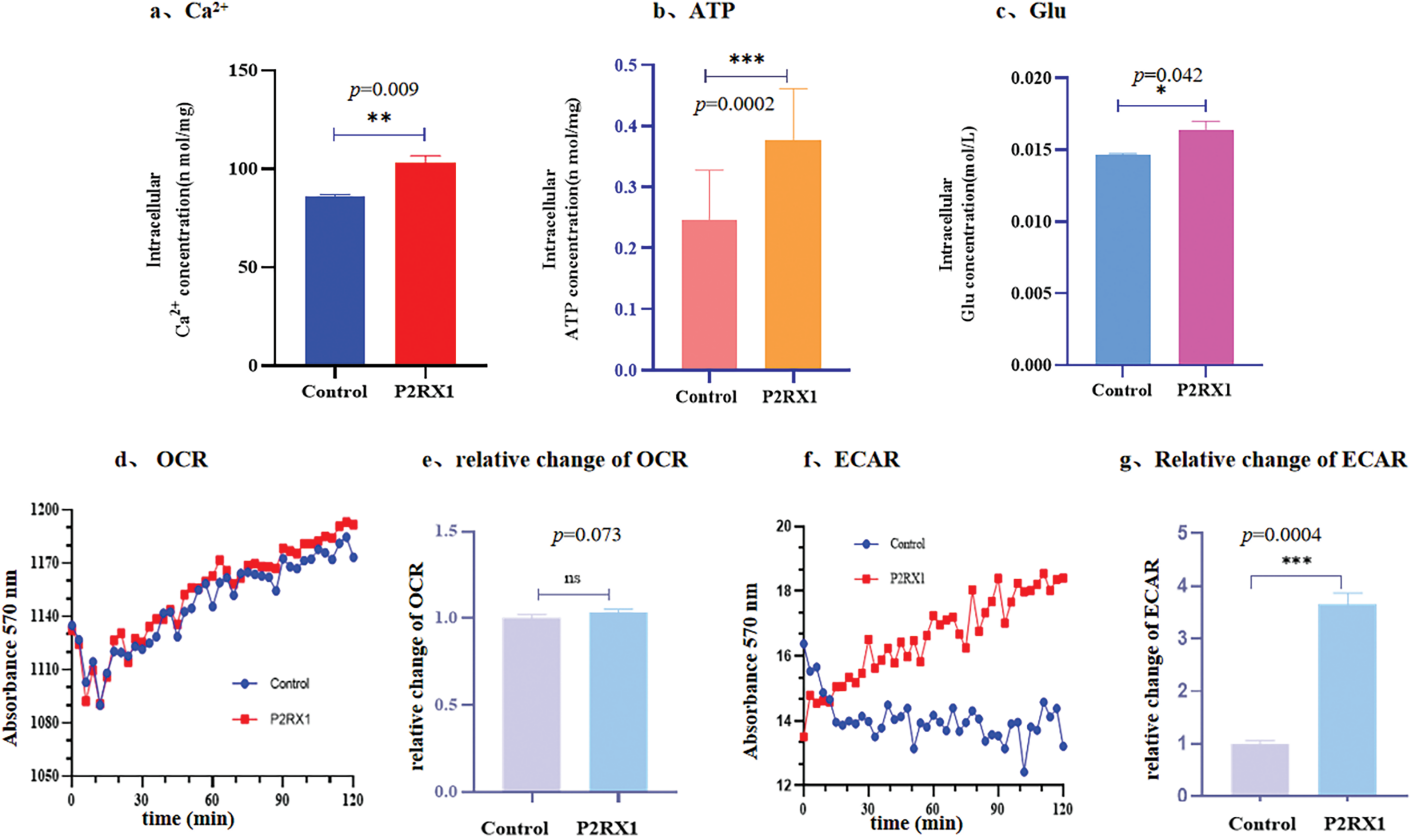
Concentration of ATP, Glu, Ca^2+^, OCR and ECAR after overexpressing P2RX1 in the intracellular. (**a**). Intracellular Ca^2+^ concentration; (**b**). Intracellular ATP concentration; (**c**). Intracellular Glu concentration; (**d**). Oxidation rate of cell consumption (OCR); (**e**). Relative change of OCR; (**f**). ECAR; (**g**). Relative change of ECAR. *p < 0.05, **p < 0.01, ***p < 0.001, ns (not significant) *p* > 0.05

To verify the above speculations, we first examined the intracellular concentrations of calcium, ATP, and glucose in P2RX1 overexpression and empty vector control SUP-B15 cells. The results showed that the intracellular concentrations of ATP, calcium ions, and Glu in the P2RX1 overexpression group were higher than those in the empty vector control group, and the differences were statistically significant (Fig. 6a–c). Second, to explore the source of high ATP in P2RX1-overexpressing SUP-B15 cells, we examined mitochondrial oxidative phosphorylation and glycolysis by measuring the cellular oxygen consumption rate (OCR) and the extracellular acidification rate (ECAR) in two groups. There were no differences in OCR (Fig. 6d,e). Moreover, the ECAR assay rate of change of the P2RX1 group was significantly higher than that of the control group (Fig. 6f,g), suggesting that the increase in SUP-B15 ATP after P2RX1 overexpression relies on the production of the glycolytic pathway rather than oxidative phosphorylation, which is in line with the theory of high glycolysis in tumor cells. The above experimental results suggest that Ph+/Ph-like ALL patients may suffer from calcium death due to persistent high intracellular calcium in tumor cells through the high expression of P2RX1, which in turn increases energy metabolism and calcium metabolism.

### 3.5 P2RX1 Gene Overexpression Inhibits Proliferation and Induces Apoptosis in SUP-B15

**Figure 7:**
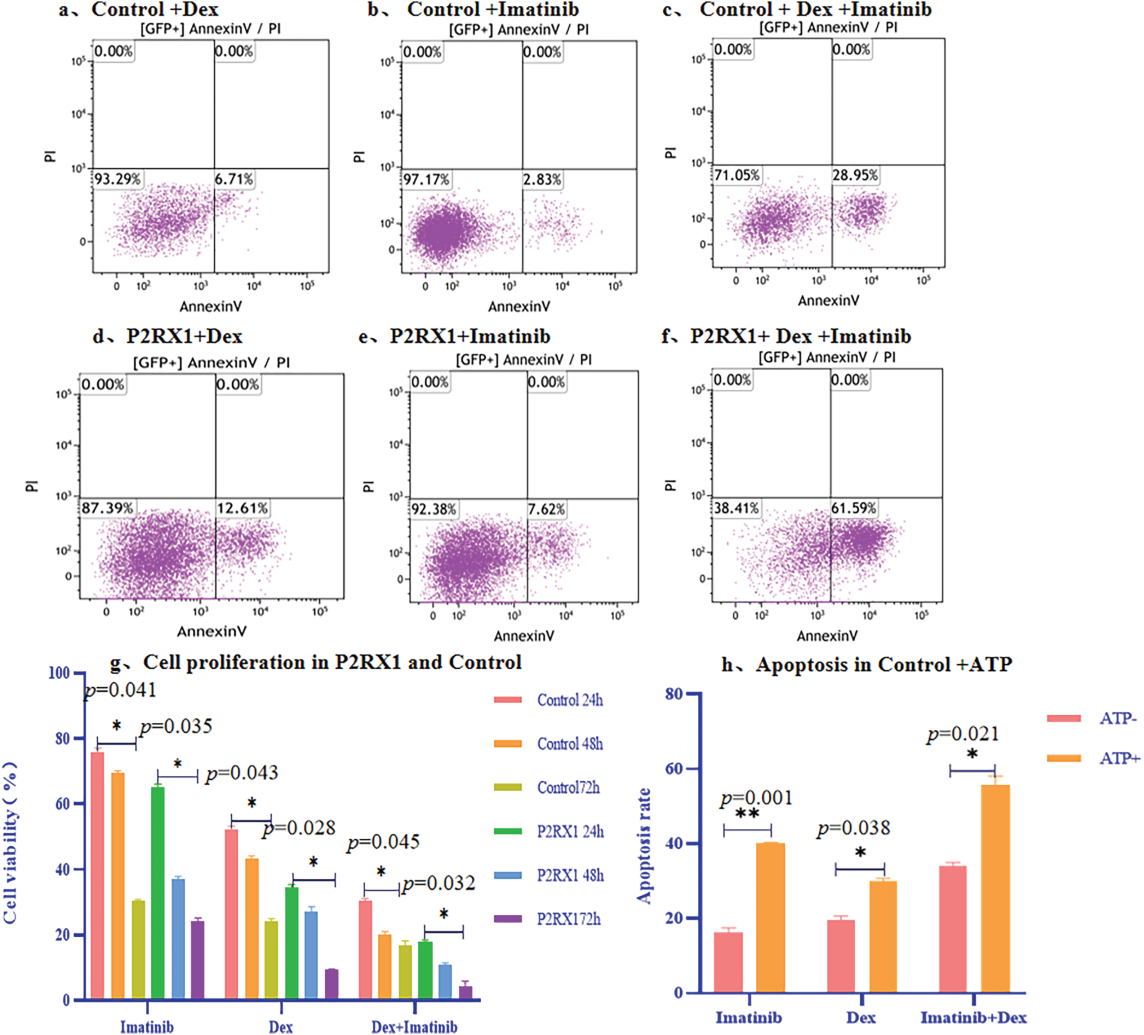
Apoptosis and proliferation of overexpression P2RX1 and Control in SUP-B15 cell. (**a**). 24 h Apoptosis of Control+Dex; (**b**). 24 h Apoptosis of Control+Imatinib; (**c**). 24 h Apoptosis of Control+Dex+Imatinib; (**d**). 24 h Apoptosis of P2RX1+Dex; (**e**). 24 h Apoptosis of P2RX1+Imatinib; (**f**). 24 h Apoptosis of P2RX1+Dex+Imatinib; (**g**). Cell proliferation in the Control and P2RX1 group; (**h**). Apoptosis of Control+ ATP. *p < 0.05, **p < 0.01

To verify the effect of P2RX1 gene expression on Ph+/Ph-like B-ALL prognosis, we cultured P2RX1 gene overexpression and null-virus-stabilized transfected SUP-B15 cell and treated them with Dex, Imatinib, and the combination of these two drugs. Apoptosis and proliferation of cells were detected after drug treatment. The results showed that the apoptosis of SUP-B15 cells increased after drug treatment in the P2RX1 group compared with the control group (Fig. 7a–f). At the same time, cell proliferation was inhibited (Fig. 7g), and the difference was statistically significant (*p* < 0.05). We simultaneously added external ATP-assisted Dex and Imatinib and combination therapy to the control group and found that apoptosis was increased (Fig. 7h), which further verified the relationship between the activation of the P2RX1 channel and the prognosis of the tumor.

**Figure 8:**
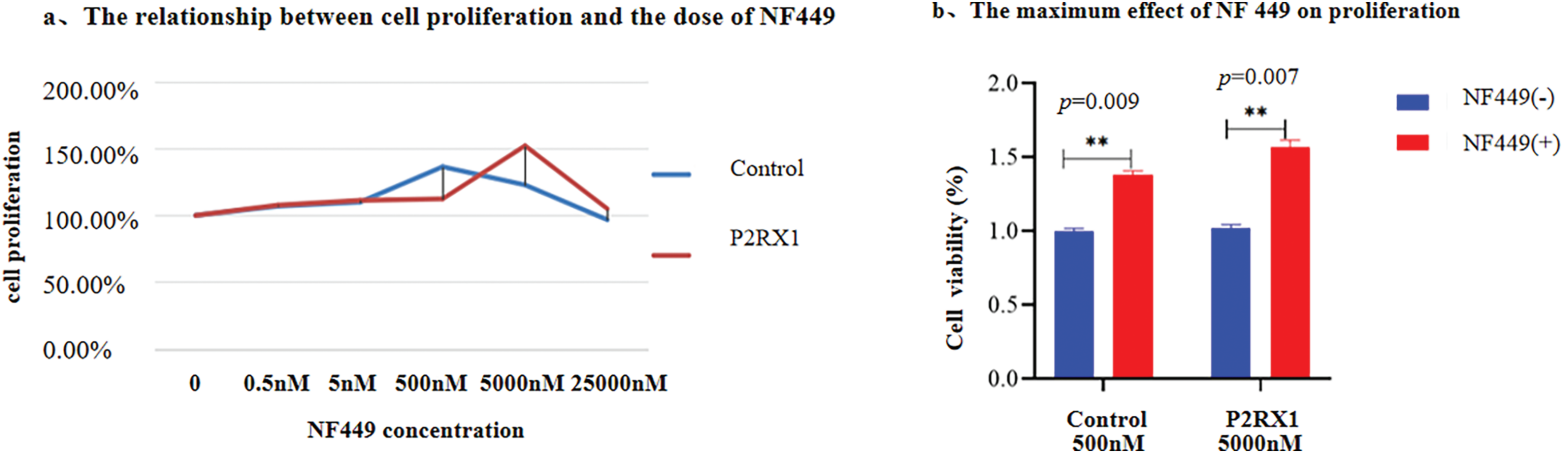
The effects of P2RX1 inhibitor NF449 on SUP-B15 cell proliferation and apoptosis. (**a**). Line graphs showing the cell proliferation ability of NC-SUP-B15 and P2RX1high SUP-B15 cells at different concentrations of NF449; (**b**). Bar chart showing the effects of (500 and 5000 nM) NF449 +Dex and Imatinib on the proliferation of NC-SUP-B15 and P2RX1high SUP-B15 cells. **p < 0.01

In addition, an inhibitor experiment was conducted. Different concentrations of P2RX1 inhibitor NF449 were used to treat P2RX1 group and control group, and the cell proliferation of the two types of cells was detected. The results showed that after NF449 inhibited the P2RX1 gene, the proliferation of both P2RX1 group and control group SUP B15 cells increased with the increase of NF449 concentration. When the NF449 concentration reached a certain level, it had a significant inhibitory effect on both groups of cells. At the same time, it was found that the tolerance of the two groups of cells to NF449 was different. The empty virus control group reached the highest cell proliferation rate at 500 nM NF449, and the P2RX1 overexpression group at 5000 nM, which were 136% and 152% respectively (Fig. 8a). The detection of cell proliferation after the cells were treated with the drug revealed that the cell proliferation was significantly inhibited. After adding (500 and 5000 nM) NF449 inhibitor, the proliferation ability of P2RX1 high SUP-B15 and NC-SUP B15 cells increased, with the control group being 75.12% and the P2RX1 overexpression group being 67.23% (Fig. 8b). These results further proved from the opposite direction that the high expression of P2RX1 inhibited the proliferation of SUPB15 cells after treatment.

### 3.6 P2RX1 via Energy Metabolism and Ca^2+^ Metabolism Affects Ph+ALL Cellular Response to Combination Therapy with Dex and Imatinib

**Figure 9:**
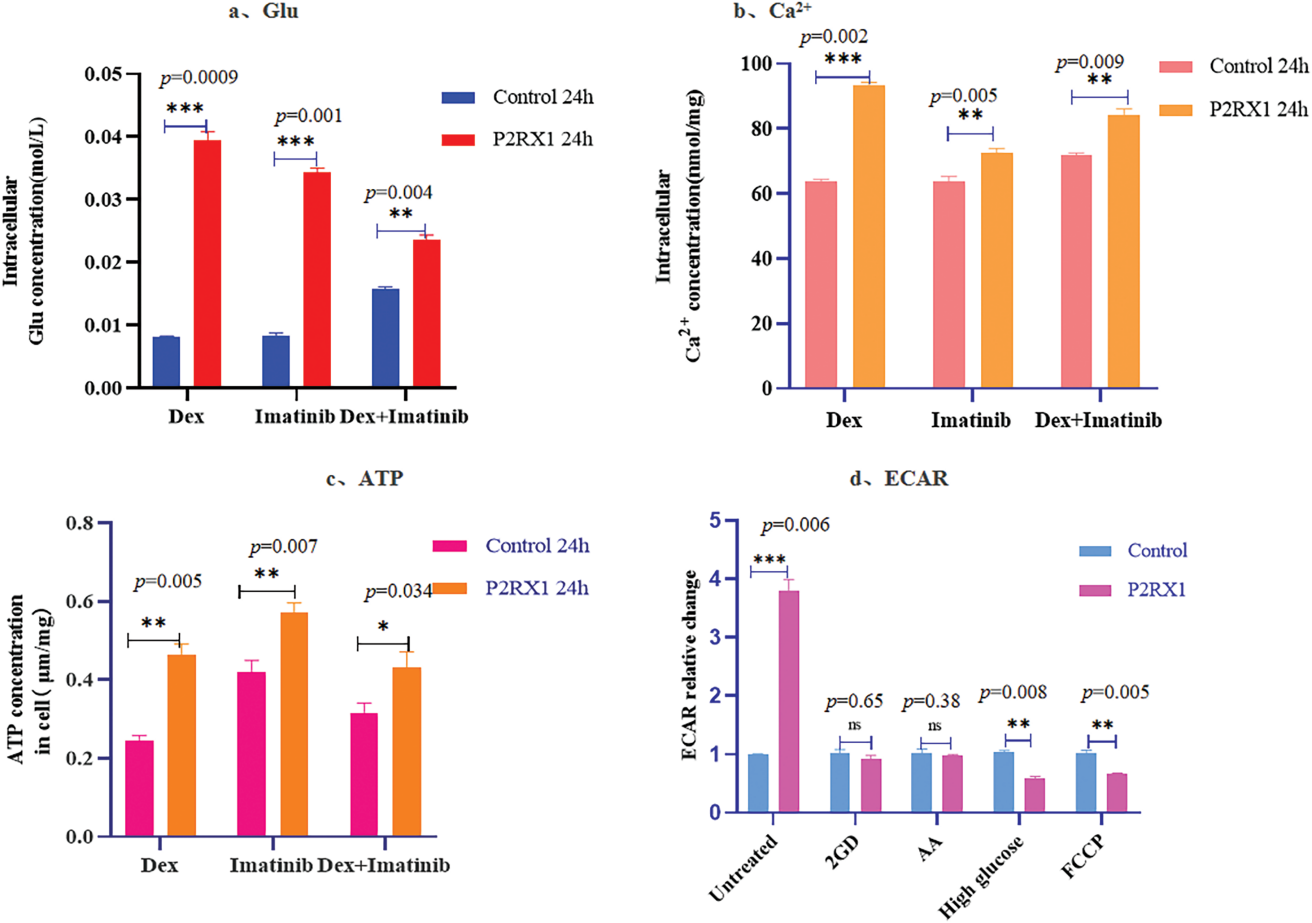
Concentration of Glu, Ca^2+^, ATP and ECAR after drug action. (**a**). Intracellular Glu concentration; (**b**). Intracellular Ca^2+^ concentration; (**c**). Intracellular ATP concentration; (**d**). Relative change of ECAR in different treatment groups. *p < 0.05, **p < 0.01, ***p < 0.001, ns (not significant) *p* > 0.05

To verify whether the P2RX1 gene affects Ph+/Ph-like ALL treatment through energy metabolism and Ca^2+^ metabolism, we examined the intracellular calcium, ATP, and glucose concentrations of both groups after examining the effects of Dex, Imatinib, and a combination of the two drugs. The results showed that the intracellular calcium ions and the concentrations of ATP and Glu were higher than those in the control group (Fig. 9a–c), and this change was more obvious in the Dex and two-drug combination treatment groups with a high apoptosis ratio, which further verified that the overexpression of the P2RX1 gene was closely related to cellular energy metabolism and Ca^2+^ metabolism. We also treated the two groups of cells using 2-GD, AA, high glucose, and FCCP, and found that FCCP and high glucose were able to correct the low rate of change of ECAR in the control group (Fig. 9d).

In summary, P2RX1 may affect the prognosis of Ph+/Ph-like ALL by influencing calcium and energy metabolism.

## 4 Discussion

Ph+/Ph-like ALL is a subtype of B-ALL with similar expression profiles, high incidence, and poor prognosis [1]. Chemotherapy, combined with TKI drugs, significantly improves the overall cure rate of Ph+/Ph-like ALL [6]. However, kinase domain mutations, especially mutations at the T315I site, significantly increase drug resistance. Although chimeric antigen receptor T (CAR-T) cell immunotherapy has a good early response rate in B-ALL, it is difficult to maintain in the long term [17]. However, bone marrow transplantation has strict requirements for donors and is difficult to popularize [18]. Therefore, finding a new therapeutic target has become the key to treatment.

The P2RX1 gene belongs to the purinergic (P2X) receptor family and encodes the P2X1 receptor—a ligand-gated ion channel—which is expressed predominantly in the cell membranes of various cell types, including smooth muscle cells, immune cells, and neuronal cells [19]. Under normal physiological conditions, P2X1 receptors are activated by extracellular nucleotides, such as adenosine triphosphate (ATP), which triggers the opening of the channel and mediates the inward flow of calcium (Ca^2+^) and sodium (Na^+^) ions and the outward flow of potassium ions (K^+^). Increased intracellular free calcium concentration is a key component in the regulation of different cellular processes, ranging from egg fertilization, active secretion, and motility to cell differentiation and death. Persistently high intracellular calcium concentrations cause calcium death of cells [20]. In disease states, aberrant expression of the P2RX1 receptor is closely associated with the development of several diseases. Numerous studies have shown that in the field of cancer, the expression level of the P2RX1 receptor is closely linked to tumor growth, metastasis, and immune escape. For example, in studying pancreatic cancer liver metastasis, it was found that P2RX1-negative neutrophil subpopulations accumulated significantly in the tumor microenvironment [21]. These cells accelerated the process of tumor metastasis by upregulating the expression of immune-suppressive molecules (e.g., PD-L1), which led to tumor cells escaping from the immune surveillance of the organism.

The overexpression of P2RX1 in gastric cancer neutrophils increases the apoptosis of cancer cells while inhibiting the migration, invasion, and viability of cells [22,23]. In lung cancer, the expression level of the P2RX1 receptor is closely related to the prognosis of patients; patients with low expression of P2RX1 often have a poor prognosis [24].

It was also found that pannexin-1 hemichannels as well as P2X1 and P2X4 receptors, promote ATP release and autocrine feedback mechanisms that control Ca^2+^ entry and T cell activation at the immunological synapse [25]. This suggests that the P2RX1 receptor may serve as a potential prognostic biomarker, providing clinicians with an important reference for evaluating patients’ conditions and formulating treatment plans.

In the present study, we found that the P2RX1 gene and the calcium metabolism and energy metabolism pathway genes in the Ph+/Ph-like ALL poor prognosis group had low expression. Cytological experiments have proved that the intracellular calcium, ATP, and Glu concentrations in P2RX1-overexpressing SUP-B15 cells were significantly higher than those in the null-viral control group. The energy metabolism assay found that cellular glycolysis was enhanced. According to Warburg, tumor cells produce energy through glycolysis even with sufficient oxygen, which is consistent with our conclusion [26].

We utilized Dex, Imatinib, and the combination of these two drugs to treat the P2RX1 overexpression SUP-B15 cell line, and found that apoptosis and proliferation inhibition in the overexpression group were significantly greater than those in the null-virus control group after the action of the drugs. Therefore, it was concluded that P2RX1 overexpression caused an increase in intracellular calcium. Simultaneously, cellular glycolysis capacity and ATP production increased, which further activated P2RX1, leading to an increase in apoptosis and proliferation inhibition. The experimental results of ATP-assisted therapy showed that external ATP supplementation could increase the apoptosis ratio and proliferation inhibition rate of SUP-B15, indicating that ATP could assist in anti-tumors.

Meanwhile, gene mutation analysis showed that there was a difference in mutated genes between the Ph+/Ph-like ALL good prognosis and poor prognosis groups, and the total number of mutant genes of the good prognosis group was higher than that of the poor prognosis group, which did not reach statistical significance, but was in line with the characteristics of the existing tumor research, i.e., a high tumor load can provide more antigenic epitopes, which is conducive to the recognition of the body’s immune system, and then enhances the killing effect of tumor cells [27–29].

In summary, low P2RX1 expression is associated with a poor prognosis of Ph+/Ph-like ALL, which may be used as an early marker of poor prognosis. Moreover, apoptosis reduction caused by low P2RX1 expression can be reversed by external ATP supplementation, ATP may assist in the combined treatment of Ph+/Ph-like ALL using Dex and Imatinib. However, this experiment also has certain shortcomings, such as a small sample size and no mouse model, among others. In the future, animal experiments will be utilized to further investigate the relationship between P2RX1 low expression and Ph+/Ph-like prognosis [30].

## Data Availability

Not applicable.

## Appendix A

**Table A1:** Primers sequence

Target genes	Forward primers (5^′^–3^′^)	Reverse primers (5^′^–3^′^)
P2RX1	GGATGGTGCTGGTACGAAACA	CACTGACACACTGCTGATAAGG
GAPDH	ATCATCAGCAATGCCTCC	CATCACGCCACAGTTTCC
MCM5	CAATGAGGAGAGGGATGTGATG	CACTCGGCAGTAGGCAATAA
